# Hypothermia for Patients Requiring Evacuation of Subdural Hematoma: A Multicenter Randomized Clinical Trial

**DOI:** 10.1007/s12028-021-01334-w

**Published:** 2021-09-13

**Authors:** Georgene W. Hergenroeder, Shoji Yokobori, Huimahn Alex Choi, Karl Schmitt, Michelle A. Detry, Lisa H. Schmitt, Anna McGlothlin, Ava M. Puccio, Jonathan Jagid, Yasuhiro Kuroda, Yukihiko Nakamura, Eiichi Suehiro, Faiz Ahmad, Kert Viele, Elisabeth A. Wilde, Stephen R. McCauley, Ryan S. Kitagawa, Nancy R. Temkin, Shelly D. Timmons, Michael N. Diringer, Pramod K. Dash, Ross Bullock, David O. Okonkwo, Donald A. Berry, Dong H. Kim

**Affiliations:** 1 The Vivian L. Smith Department of Neurosurgery, McGovern Medical School, University of Texas Health Science Center at Houston, 6431 Fannin Street, MSB 7.156, Houston, TX 77030 USA; 2 Memorial Hermann Hospital, Texas Medical Center, Houston, TX USA; 3 Department of Emergency and Critical Care Medicine, Nippon Medical School Hospital, Tokyo, Japan; 4 Statistical and Software Team, Berry Consultants, Austin, TX USA; 5 Department of Neurological Surgery, University of Pittsburgh Medical Center, Pittsburgh, PA USA; 6 Department of Neurological Surgery, Jackson Memorial Hospital, University of Miami, Miami, FL USA; 7 Department of Emergency, Disaster, and Critical Care Medicine, Kagawa University Hospital, Kagawa Prefecture, Japan; 8 Emergency and Critical Care Medicine, Kurume University Hospital, Fukuoka, Japan; 9 Department of Neurosurgery, Yamaguchi University Hospital, Yamaguchi, Japan; 10 Department of Neurological Surgery, Grady Memorial Hospital, Emory University School of Medicine, Atlanta, GA USA; 11 H. Ben Taub Department of Physical Medicine and Rehabilitation, Baylor College of Medicine, Houston, TX USA; 12 Departments of Neurological Surgery and Biostatistics, University of Washington, Seattle, WA USA; 13 Department of Neurological Surgery, Indiana University Health, Indiana University School of Medicine, Indianapolis, IN USA; 14 Departments of Neurology, Neurological Surgery, Anesthesiology, and Occupational Therapy, Washington University School of Medicine, St. Louis, MO USA; 15 Department of Neurobiology and Anatomy, McGovern Medical School, The University of Texas Health Science Center at Houston, Houston, TX USA

**Keywords:** Hypothermia (induced), Hematoma (subdural), Brain injuries (traumatic)

## Abstract

**Background:**

Hypothermia is neuroprotective in some ischemia–reperfusion injuries. Ischemia–reperfusion injury may occur with traumatic subdural hematoma (SDH). This study aimed to determine whether early induction and maintenance of hypothermia in patients with acute SDH would lead to decreased ischemia–reperfusion injury and improve global neurologic outcome.

**Methods:**

This international, multicenter randomized controlled trial enrolled adult patients with SDH requiring evacuation of hematoma within 6 h of injury. The intervention was controlled temperature management of hypothermia to 35 °C prior to dura opening followed by 33 °C for 48 h compared with normothermia (37 °C). Investigators randomly assigned patients at a 1:1 ratio between hypothermia and normothermia. Blinded evaluators assessed outcome using a 6-month Glasgow Outcome Scale Extended score. Investigators measured circulating glial fibrillary acidic protein and ubiquitin C-terminal hydrolase L1 levels.

**Results:**

Independent statisticians performed an interim analysis of 31 patients to assess the predictive probability of success and the Data and Safety Monitoring Board recommended the early termination of the study because of futility. Thirty-two patients, 16 per arm, were analyzed. Favorable 6-month Glasgow Outcome Scale Extended outcomes were not statistically significantly different between hypothermia vs. normothermia groups (6 of 16, 38% vs. 4 of 16, 25%; odds ratio 1.8 [95% confidence interval 0.39 to ∞], *p* = .35). Plasma levels of glial fibrillary acidic protein (*p* = .036), but not ubiquitin C-terminal hydrolase L1 (*p* = .26), were lower in the patients with favorable outcome compared with those with unfavorable outcome, but differences were not identified by temperature group. Adverse events were similar between groups.

**Conclusions:**

This trial of hypothermia after acute SDH evacuation was terminated because of a low predictive probability of meeting the study objectives. There was no statistically significant difference in functional outcome identified between temperature groups.

**Supplementary Information:**

The online version contains supplementary material available at 10.1007/s12028-021-01334-w.

## Introduction

Nearly 2.88 million Americans sustain a traumatic brain injury (TBI) annually, and of them 56,800 die and 90,000 remain permanently disabled [1, 2]. Brain damage as a result of TBI is caused by the primary injury and multiple secondary pathological processes that occur as a result of the initial trauma [3]. Although there is no cure for the primary injury at present, there is ongoing work to develop neuroprotective treatments to prevent and attenuate secondary injury. Early hypothermia is one modality shown to be neuroprotective in ischemia–reperfusion injuries, such as in preclinical TBI models [4, 5], after cardiac arrest [6–8], and in infants with hypoxic-ischaemic encephalopathy [9, 10]. However, clinical trial results on the effect of hypothermia on outcome after TBI have not proven efficacy [11–13].

Retrospective subgroup analysis of the National Acute Brain Injury Study: Hypothermia I [14] and II [15] hypothermia trials revealed that patients with TBI who were treated with hypothermia undergoing surgical evacuation of intracranial hematomas had significantly improved neurologic outcomes compared with patients treated with normothermia [16]. We hypothesized that therapeutic hypothermia, when applied to a more homogeneous population of patients after subdural evacuation, would improve clinical outcomes by preventing secondary pathological processes from ischemia–reperfusion injury.

We performed a randomized controlled prospective trial to study the effect of early therapeutic hypothermia in patients undergoing surgical evacuation of acute subdural hematomas (SDH), called “Hypothermia for Patients requiring Evacuation of Subdural Hematoma: a Multicenter, Randomized Clinical Trial,” the “HOPES Trial.” The primary objective of the study was to determine whether rapid induction of hypothermia prior to emergent craniotomy for traumatic SDH would improve outcome, as measured by Glasgow Outcome Scale Extended (GOSE) at 6 months. Secondary objectives were to assess the safety of intravascular cooling in the management of SDH and to explore the effect of hypothermia on TBI plasma biomarkers, glial fibrillary acidic protein (GFAP) and ubiquitin C-terminal hydrolase L1 (UCH-L1) [17, 18]. GFAP, an astroglia marker, and UCH-L1, a neuronal marker, are known to be elevated within hours after TBI [19–21]. GFAP and UCH-L1 have successfully detected lesions visible on head computed tomography [19].

## Methods

### Participants

This was a prospective, pragmatic, randomized, controlled, multicenter trial to assess the safety and efficacy of intravascular cooling to induce hypothermia in patients with TBI prior to and after surgical evacuation of SDH. The trial enrolled adult (22–65 years of age) patients with TBI within 6 h of SDH who were not following commands (Glasgow Coma Scale [GCS] motor score ≤ 5). Patients were excluded if there was no planned evacuation of the SDH, concomitant injury or history contraindicated hypothermia for patient safety, arrival temperature was < 35 °C, total GCS = 3 and the patient had fixed and dilated pupils, or there was an inability to obtain consent or use the exception to informed consent for emergency research. Investigators at tertiary care medical centers in the United States and Japan enrolled patients under institutional review board approved protocols.

### Intervention

Patients were randomly assigned into two groups. The intervention group received rapid induction of hypothermia to 35 °C followed by maintenance at 33 °C for 48 h up to 5 days. Rewarming occurred at a rate of 0.25 °C/h. Cooling and rewarming interventions were based on recommendations from Clifton et al. [15]. If intracranial pressure (ICP) increased during rewarming temperature was held constant, and rewarming resumed after standard ICP control measures were instituted. The control group received standard care, including temperature maintenance at normothermia (37 °C) for 48 h. Temperature variations of ± 0.5 °C were permissible. Warming of control patients prior to surgery is per standard care. Standard care for rewarming is to warm patients slowly, based on prior indications of poor outcome due to rapid rewarming [14, 22]. Intravascular catheters (Thermogard XP System with Quattro catheter; ZOLL Circulation Inc, San Jose, CA) and fever control were used for hypothermia and to maintain normothermia, as it has been established that fever is detrimental in patients with TBI [23–25]. Intravascular temperature management has previously been shown to reach the target temperature rapidly and safely [26–28]. Shivering was managed according to an established protocol used by the intensive care units [29]. Other care was at the discretion of the treating physicians. All centers’ practice incorporated the Brain Trauma Foundation Guidelines [30].

### Outcomes

Neuropsychological assessment of patients’ level of recovery was performed at 4 weeks and 6 months post-injury by investigators who were unaware of the treatment group assignment. Dichotomized GOSE at 6 months post-injury was the primary outcome [31]. Good recovery and moderate disability were designated as favorable outcomes; and severe disability, vegetative state, and death were designated as poor outcomes. GOSE is a widely used global outcome score with good interater and intrarater reproducibility [31]. GOSE assesses consciousness, independence, work status, and return of lifestyle via a structured interview.

### Design

The trial design aimed for *N* = 120 patients and allowed for an extension up to 350 patients. The design included multiple interim analyses after 60, 120, 180 and 240 patients were randomized. At each interim analysis, Bayesian predictive probabilities were to be used to determine whether enrollment should stop for either success or futility before the maximum enrollment. The operating characteristics of the study design (type I error, power, and expected sample size) were computed by simulation in a variety of possible scenarios. Treatment effect was based on data from Clifton et al. [16]. The trial had power greater than 90% for a scenario where the proportion of 6-month GOSE score is good in the treatment arm was 0.604 compared to 0.347 in the control arm and the trial was quite likely to stop at or before the *N* = 120 interim analysis for success. Type I error was controlled at 0.025 and the trial was quite likely to stop for futility at or before the *N* = 120 interim analysis.

Due to slow accrual, an early futility interim analysis was added to occur after *N* = 31 participants had completed 6-month follow-up. Enrollment continued during this analysis, but decisions would be based on the data for the initial 31 patients. This analysis computed the probability of success (probability of statistically significantly higher rate of favorable GOSE in the hypothermia arm) after enrollment of 60 patients, which was then viewed as the absolute maximum feasible sample size given the slow accrual. Predetermined stopping guidelines dictated that if the predictive probability of success with a sample size of 60 patients was less than 0.40, the trial was to stop for futility. This predictive probability was calculated based on Beta-Binomial distributions and used non-informative Beta (0.5, 0.5) prior distributions on the parameters. Results were provided only to the DSMB who gave a recommendation regarding trial continuance based on the totality of the evidence.

### Randomization

To reduce the likelihood of imbalance of important prognostic factors between centers the study used a blocked randomization scheme that randomized equally at a 1:1 ratio to hypothermia versus control normothermia. Randomization was generated using a computer program by the independent statistical team who provided sites sequentially numbered sealed opaque envelopes. Once eligibility was confirmed site study physicians and nurses enrolled participants, opened the randomization envelope and assigned the participants to the designated intervention.

### Blinding

Participants and clinical care providers were not blind to assignment. Investigators were unaware of treatment assignments and outcomes of other sites’ patients. Outcomes assessors were blinded to the patients’ treatment arm.

### Enzyme-Linked Immunosorbent Assays

Investigators used enzyme-linked immunosorbent assays (ELISA) to evaluate the plasma levels of GFAP and UCH-L1 on the 24 patients with blood samples (13 hypothermia, 11 normothermia) that were available at three time points. Time 1 (T1) was collected at less than 6 h post TBI and precooling. T2 was collected 6 to 48 h post TBI (within the time of temperature management); and T3 was collected 5 to 14 days post TBI (post cooling). Control standards and samples from six healthy volunteers were measured. Blood was collected in K2 EDTA vacutainer tubes (BD, Franklin Lakes, NJ), processed within an hour of draw and frozen at − 80 °C. The concentration of GFAP was measured using a sandwich enzyme immunoassay (BioVendor, Ashville, NC) according to manufacturer’s instructions. The lower limit of detection for this assay is.045 ng/mL. UCH-L1 sandwich ELISAs were developed using the UCH-L1 DuoSet ELISA (R&D Systems, Minneapolis, MN). The lower limit of detection for the UCH-L1 ELISA was 19.5 pg/mL. All samples were analyzed in duplicate.

### Adverse Events

A secondary objective was to evaluate the safety of intravascular cooling in the management of acute traumatic SDH. Adverse events were monitored for the 6-month study period. Serious adverse events were graded according to the United States Department of Health and Human Services (USDHHS) Common Terminology Criteria for Adverse Events V4.0 [32]. Predefined adverse events of interest that were of concern with hypothermia treatment were selected to be monitored and reported regardless of grade. These included cardiac arrhythmias, thromboembolic events, pneumonia, bleeding/hemorrhage, infection (culture positive, e.g., blood stream infection, urinary tract infection, ventriculitis), and death.

An independent DSMB consisting of a neurosurgeon, a critical care physician and a statistician all of whom are experts in the fields of TBI and hypothermia monitored the study. The study was registered on ClinicalTrials.gov as NCT02064959 and UMIN000014863 in Japanese UMIN Clinical Trials Registry. The exception to informed consent for emergency research provision was used if permitted by the local institutional review board and local law. Berry Consultants performed the statistical design and independent statistical analysis.

### Statistical Methods

The analysis was performed using a modified intent-to-treat population of all randomly assigned patients having no exclusion criteria. Demographic and baseline data were summarized using means, standard deviations, or count and percentage. Adverse events are reported by type, temperature group and frequency. Descriptive data comparing groups were tested for normality using the Shapiro–Wilk test and analyzed using Student’s *t*-test and reported as means and standard deviation, or if not normally distributed, using the Mann–Whitney Rank Sum test and reported as medians and interquartile ranges (IQR). A one-sided Fisher’s exact test with a predefined significance level of *p* < 0.02 to compare proportions of patients with good outcomes as measured by GOSE at 6 months is reported as the primary outcome. ELISA data were log transformed to normalize values and analyzed using two-way repeated measures analysis of variance (ANOVA). The ELISA analyses were exploratory, and significance was assessed at the.05 level.

## Results

**Fig. 1:**
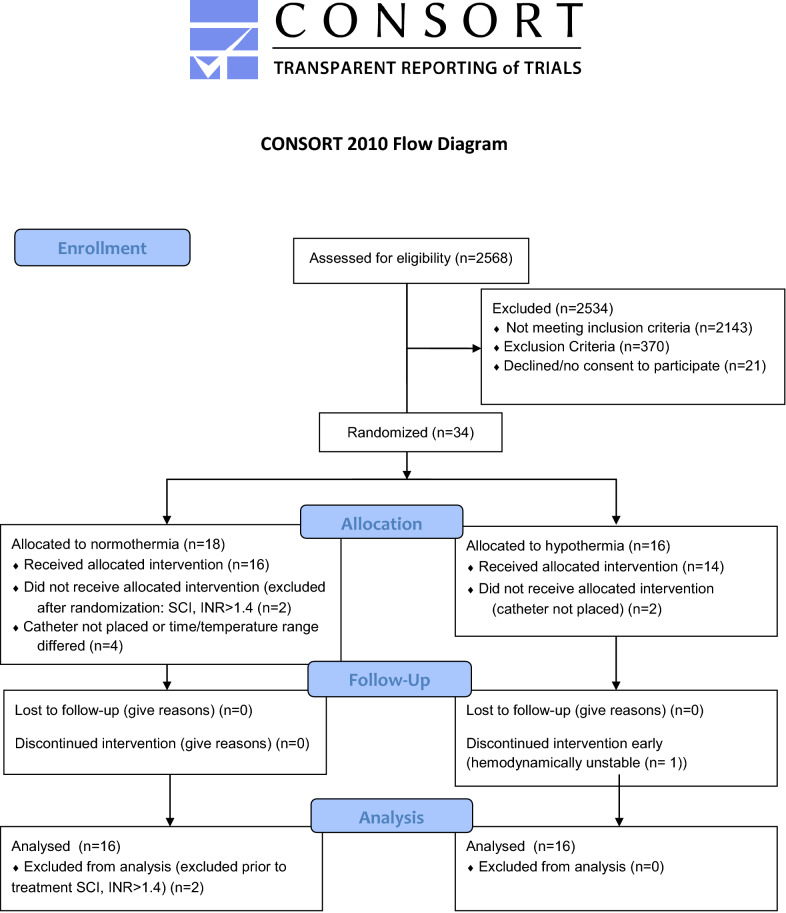
CONSORT flow diagram. Investigators screened 2568 patients for study eligibility. Of these, 2534 were excluded for not meeting enrollment criteria. This included 2143 who did not meet inclusion criteria: there was no SDH or no evacuation planned (*n* = 749), age > 65 years or < 22 years (*n* = 956), arrival was outside of time window (*n* = 231) or the patient was following commands (*n* = 207). Additionally, 370 people met exclusion criteria by having GCS = 3, fixed and dilated pupils or duret hemorrhage (*n* = 144), known preexisting neurological deficit (*n* = 103), other contraindication to hypothermia (*n* = 108), arrival temperature < 35 °C (*n* = 6), spinal cord injury (*n* = 4), prisoner (*n* = 4), pregnant (*n* = 1), or an inability to obtain consent, use exception from informed consent or declined to participate (*n* = 21). GCS, Glasgow Coma Scale; INR, international normalize ratio; SDH, subdural hematoma

**Fig. 2:**
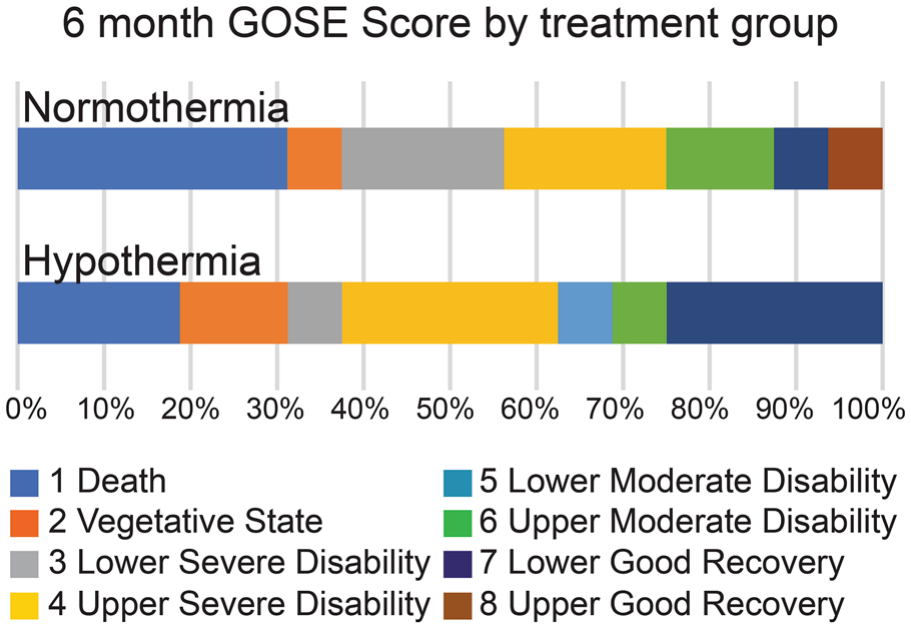
Six-month GOSE score by treatment group. Post hoc analysis of the percentage of patients by treatment group within each GOSE grade. Primary outcome analysis separated favorable from unfavorable between Grade 4, upper severe disability and Grade 5, lower moderate disability

Patients (*n* = 34) were recruited between May 2014 and July 2018, two were excluded from the study after randomization, but prior to intervention because they met exclusion criteria (Fig. 1). Enrolled patients (*n* = 32) were followed for 6 months with the last patient completing follow-up in February 2019. The trial was stopped for futility after interim analysis of 31 patients showed that the trial would not be likely to reach a decisive outcome when 60 patients completed the study (predictive probability of success at *N* = 60 was 1.64%). After the trial ended and data were cleaned, one participant’s 6-month outcome was corrected and the last patient’s outcome was included. Note that the predictive probability calculation was performed prior to finalization of data. Favorable GOSE at 6 months did not differ between hypothermia and control groups (6/16, 38% vs. 4/16, 25%; odds ratio [OR] of 1.8, 95% confidence interval [CI] 0.39 to ∞, *p* = 0.35, respectively). Figure 2 illustrates ordinal data of 6-month GOSE outcome by treatment group (Fig. 2). A post hoc analysis adjusting for key covariates (age, GCS motor score and pupillary response) did not change the study results.

Demographics are similar between treatment groups (Table 1). All patients had a GCS motor score < 6 (not following commands) and were classified as moderate to severe TBI. SDH was present in all patients and hematoma volumes and midline shift on the presurgical head computed tomography scan were not different between normothermia and hypothermia groups.

**Table 1:** Demographics

Descriptor	Normothermia control (*n* = 16)	Hypothermia (*n* = 16)	Total (*n* = 32)	
Sex				
Male	14	11	25 (78%)	
Female	2	5	7 (22%)	
Average age, SD (yr)	41.2 ± 12.7	46.6 ± 15.1	43.9 ± 14.0	
Ethnicity				
Hispanic	3	4	7 (22%)	
Not Hispanic	13	11	24 (75%)	
Unknown	0	1	1 (3%)	
Race				
White	10	10	20 (63%)	
Asian	5	4	9 (28%)	
Black/African American	1	2	3 (9%)	
Average height (cm)	172 ± 7.1	168.5 ± 13.3	170.2 ± 10.6	
Average weight (kg)	80.4 ± 19.9	77.2 ± 18.1	78.8 ± 18.8	
GCS score on arrival	6.2 ± 2.6	6.5 ± 2.1	6.3 ± 2.3	
Hematoma volume (cm^3^)	50.5 ± 23.6	45.9 ± 24.8	48.2 ± 23.9	
Midline shift	9.2 ± 4.9	7.8 ± 3.9	8.5 ± 4.4	
Diffuse axonal injury				
Present	4 (25%)	2 (13%)	6 (19%)	
Indeterminate	3 (19%)	3 (19%)	6 (19%)	
Absent	9 (56%)	10 (63%)	19 (59%)	
Not documented	0	1 (3%)	1 (3%)	
Abnormal pupils	8 (50%)	9 (56%)	17 (53%)	
Time to hypothermia induction or reaching normothermia (h)	4.0 ± 3.1	4.8 ± 2.5	4.3 ± 2.9	

*GCS* Glasgow coma scale, *SD* standard deviation

Temperature was not different between groups on arrival (36.7 °C [IQR 36.15–36.98 °C] hypothermia group and 36.15 °C [IQR 35.70–36.78 °C] normothermia group, *p* = 0.2). Temperature at the time of dura opening was lower for the hypothermia group 35 °C (IQR 34.99–35.08 °C) compared with 35.95 °C (IQR 35.1–36.5 °C) for the normothermia group (*p* = 0.004). Four patients were unable to have the venous catheter placed prior to surgery; two of these were in the hypothermia group. The treating physicians decided not to cool these two patients. One of them did not reach 35 ± 0.5 °C at the time of the dura opening. Six patients in the normothermia group were within the hypothermia temperature range (< 35.5 °C) at the time of dura opening and were being warmed per standard care per protocol. Treating physicians actively rewarmed one patient who was hemodynamically unstable at 24.8 h instead of 48 h.

### Ancillary Analyses

**Fig. 3:**
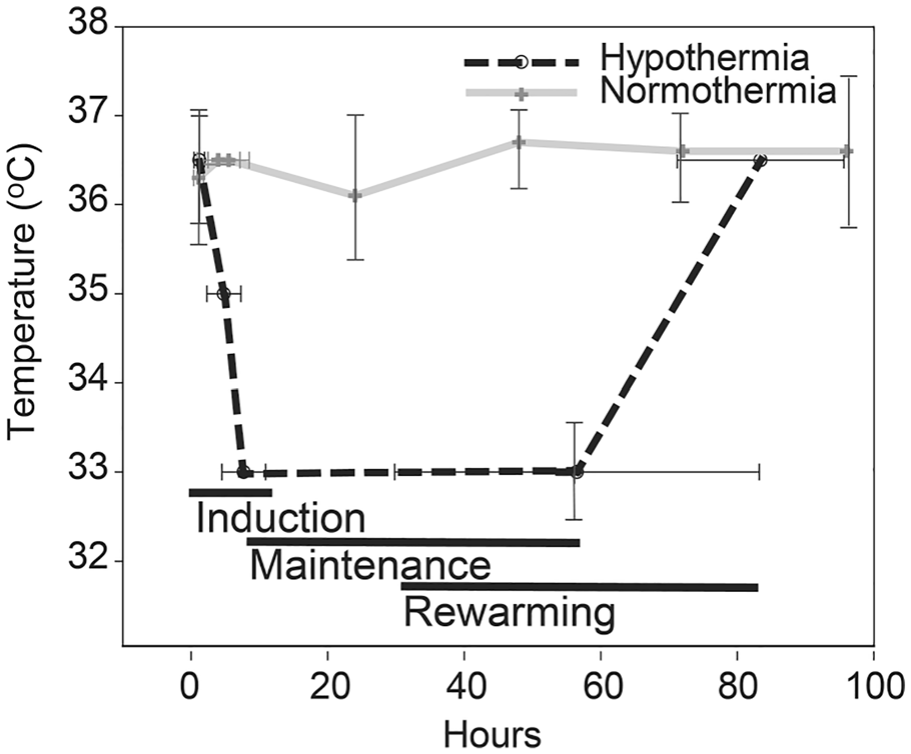
Variance in time for therapeutic temperature management. Figure 3 displays the time variance from injury to induction of hypothermia, maintenance, and rewarming. The graph represents the 14 of 16 patients with hypothermia who had catheters placed and were treated with hypothermia and 16 of 16 patients with normothermia. The temperature of the hypothermia treatment group is compared to the mean daily low temperature of the normothermia group

There were 14 of 16 patients managed per intent at hypothermia. Figure 3 displays the variance of time from injury to hypothermia induction, maintenance and rewarming compared to the mean daily low temperature of the normothermia group (Fig. 3).

Intensive care and hospital length of stay data were available for all but one patient with normothermia. Intensive care unit length of stay did not differ between groups, 12.95 days (IQR 9.2–17.7) hypothermia versus 11.4 days (IQR 7.95–32.4) normothermia (*p* = 0.7). Hospital length of stay was not different between hypothermia and normothermia groups 20.7 (IQR 17.8–29.8) versus 18.2 (IQR 8.2–45.8) days, *p* = 0.9, respectively.

To examine whether differences in clinical variables explain the lack of differences in outcome in our treatment groups, post hoc analysis of blood pressure, ICP, blood gases, and blood glucose levels was performed. Daily high and low mean arterial pressure (MAP), mean partial pressure of carbon dioxide, mean ICP, and mean blood glucose levels were examined by treatment group over 8 days post TBI. We observed no group differences in these clinical variables (Supplementary Fig. 1).

### Harms

Adverse events are summarized as serious adverse events (Table 2) and other, not serious adverse events (Table 3). There was no difference in the number of adverse events per participant by treatment group, 4.5 (IQR 25–9) events per normothermia participant versus 4 (IQR 2–7.8) events per hypothermia participant (*p* = 0.7). In the normothermia group, 5 of 16 (31%) patients experienced new or increased bleeding. These included one with worsening swelling and hemorrhage during surgery and, post-operatively, two with epidural hematomas, one with intracranial hemorrhage, and one with a worsening contusion. There was no new or increased bleeding observed in the hypothermia treatment group.

**Table 2:** Serious adverse events

Adverse event term	Organ system	Normothermia, count	Hypothermia, count	Total, count
Anemia	Blood and lymphatic	6	4	10
Elevated white blood cell count	Blood and lymphatic	0	1	1
Sinus bradycardia	Cardiac	1	1	2
Tachycardia, agitation	Cardiac	1	0	1
Infection	GI	0	2	2
Mesenteric ischemia with lactic acid disorder (elevated)	GI	0	1	1
Cholecystitis, acute	Hepatobiliary	1	0	1
Acidosis	Metabolism and nutrition	0	1	1
Hyperglycemia	Metabolism and nutrition	1	0	1
Hypermagnesemia	Metabolism and nutrition	1	4	5
Hypernatremia	Metabolism and nutrition	4	1	5
Hypocalcemia	Metabolism and nutrition	1	0	1
Hypokalemia	Metabolism and nutrition	3	1	4
Hypophosphatemia	Metabolism and nutrition	4	2	6
Death	Nervous	5	3	8
Epidural hematoma	Nervous	2	0	2
Hospital readmission/facial droop	Nervous	0	1	1
Hydrocephalus	Nervous	2	0	2
Elevated intracranial pressure	Nervous	2	0	2
Intracranial hemorrhage	Nervous	1	0	1
Muscle weakness upper limb	Nervous	0	1	1
Neurological worsening	Nervous	0	1	1
Seizure	Nervous	1	0	1
Stroke	Nervous	5	1	6
Swelling and hemorrhage during surgery	Nervous	1	0	1
Worsening contusion	Nervous	1	0	1
Urinary tract infection	Renal and urinary	2	1	3
Laryngeal oedema	Respiratory	1	0	1
Acute respiratory distress syndrome	Respiratory	1	0	1
Hospital readmission/chest wall hematoma	Respiratory	1	0	1
Pneumonia	Respiratory	4	5	9
Pneumothorax	Respiratory	1	0	1
Sepsis	Respiratory	0	1	1
Vascular access complication	Surgical and medical procedures	0	1	1
Hypotension	Vascular	0	1	1
Thromboembolic event	Vascular	5	4	9

Serious adverse events were graded according to the USDHHS CTCAE V4.0. CTCAE grade 3 or higher were classified as severe adverse events

CTCAE, Common Terminology Criteria for Adverse Events, GI, gastrointestinal system, USDHHS, United States Department of Health and Human Services

**Table 3:** Other nonserious events

Adverse event term	Organ system	Normothermia, count	Hypothermia, count	Total, count
Anemia	Blood and lymphatic	4	4	8
CPK increased	Blood and lymphatic	1	0	1
Lymphocyte decreased	Blood and lymphatic	1	0	1
Sinus bradycardia	Cardiac	1	2	3
Supraventricular tachycardia	Cardiac	0	1	1
Fever	General	1	1	2
Constipation	GI	0	1	1
Diarrhea	GI	0	1	1
Blood bilirubin increased	Hepatobiliary	1	0	1
Liver dysfunction	Hepatobiliary	0	1	1
Lipase increased	Investigations	1	0	1
Acidosis	Metabolism and nutrition	0	1	1
Alkalosis	Metabolism and nutrition	1	1	2
Hypermagnesemia	Metabolism and nutrition	1	2	3
Hypernatremia	Metabolism and nutrition	2	2	4
Hypokalemia	Metabolism and nutrition	1	2	3
Hyponatremia	Metabolism and nutrition	0	1	1
Hypophosphatemia	Metabolism and nutrition	2	4	6
Wound infection	Musculoskeletal and connective tissue	0	1	1
Cranioplasty	Nervous	0	1	1
Hydrocephalus	Nervous	1	0	1
Seizure	Nervous	2	2	4
Brain abscess	Nervous	0	1	1
Meningitis	Nervous	0	1	1
Neurological worsening	Nervous	1	0	1
Pneumonia	Respiratory	3	6	9
Laryngeal oedema	Respiratory, thoracic, and mediastinal	0	1	1
Drug eruption	Skin and subcutaneous	0	1	1
Scalp wound/infection	Skin and subcutaneous	0	1	1
Wound drainage	Skin and subcutaneous	0	1	1
Replacement of catheter	Surgical and medical procedures	0	1	1
Hypotension	Vascular	0	1	1
Thrombus (superficial)	Vascular	1	0	1

Specific predefined adverse events of interest that historically were known to be of concern with hypothermia treatment were selected to be monitored and reported regardless of grade. Those that were less than USDHHS CTCAE V4.0 grade 3 and other reported nonserious events are listed

CPK, Creatine phosphokinase, CTCAE, Common Terminology Criteria for Adverse Events, GI, gastrointestinal system, USDHHS, United States Department of Health and Human Services

### ELISA

**Fig. 4:**
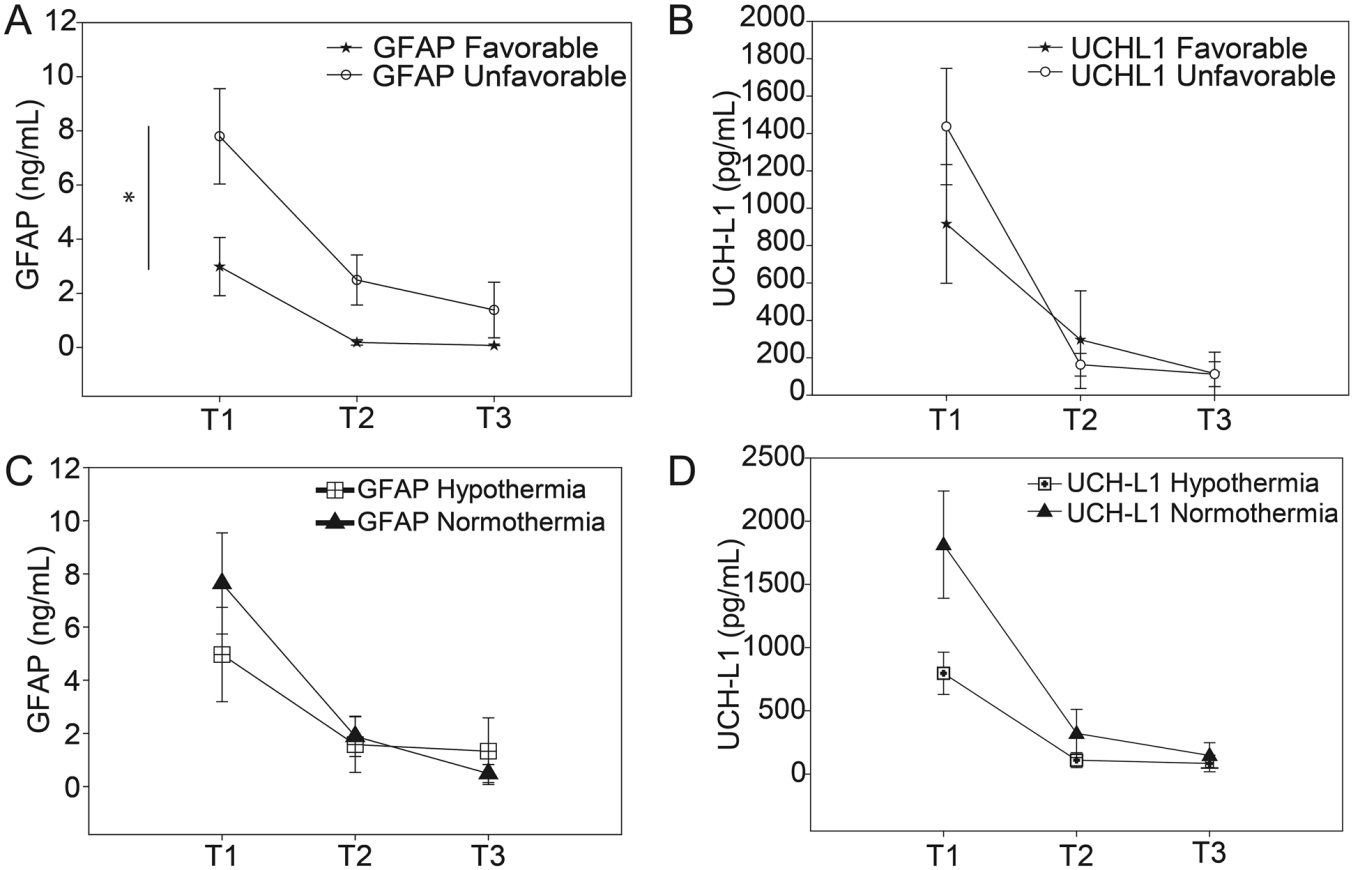
Plasma GFAP and UCH-L1 levels over time by outcome group and by temperature group. **a** Plasma levels of glial fibrillary acidic protein (GFAP) and ubiquitin C-terminal hydrolase L1 (UCH-L1) were measured by enzyme-linked immunosorbent assay (ELISA). T1 GFAP levels were elevated compared with T2 and T3 and patients with favorable outcome had significantly lower GFAP levels than those with unfavorable outcome, *p* < .04. **b** T1 UCH-L1 levels were higher than T2 and T3 UCH-L1 levels, but no difference in UCH-L1 was detected between outcome groups, *p* = .26. When samples were separated by temperature treatment groups the markers were elevated at T1 compared with T2 and T3. However, there was no difference detected between hypothermia and normothermia treatment groups for GFAP levels, *p* = 0.28 (**c**) or UCH-L1 levels, *p* = 0.46 (**d**)

At T1, median plasma GFAP and UCH-L1 levels of patients with SDH were elevated compared to healthy controls. GFAP levels at T1 were 3.39 (IQR 1.35–8.66) ng/mL compared with 0 (IQR 0–0.12) ng/mL for healthy comparators (*p* < 0.001). UCH-L1 levels at T1 were 1.07 (IQR 39–1.82) ng/ml compared to 0 ng/mL for healthy comparators (*p* < 0.001). When separating the patients by outcome group, two-way repeated measures ANOVA indicated that GFAP (*p* = 0.036) [but not UCH-L1 (*p* = 0.26)] levels were lower in the patients with favorable outcome compared with those with unfavorable outcome. The T1 samples showed higher levels of these biomarkers as compared to the levels in T2 or T3 samples (Fig. 4a GFAP and b UCH-L1). Separating the biomarker results by temperature group, plasma levels of both GFAP and UCH-L1 were elevated within the first 6 h of injury (T1) and were significantly higher at T1 compared with T2 and T3. However, two-way ANOVA analysis indicated that neither marker differed by temperature group (Fig. 4c and d). Thus, an effect from the hypothermia treatment on GFAP and UCH-L1 levels cannot be identified in these samples.

## Discussion

The Hypothermia for Patients requiring Evacuation of Subdural Hematoma study was terminated early due to futility. An interim analysis for futility resulted in a predictive probability of trial success once 60 patients have 6-month GOSE outcomes of 1.64%, below the prespecified threshold indicating the trial should stop for futility. The final analysis of 32 patients completing their 6 months follow-up showed that a significant difference in GOSE at 6 months between treatment groups could not be detected.

### Outcomes

Our statistical design planned for enrollment of 120 patients. Therefore, conclusions on the 32 patients must be interpreted with the understanding that our sample size is a limitation. A recent meta-analysis of severe TBI hypothermia trials that utilized protocols similar to the one used in the present study indicated a reduced death rate in those treated with hypothermia (33–35 °C) compared with no cooling (OR = 0.627, *p* = 0.05) [33]. Although we also had fewer deaths in the hypothermia group, the number of those surviving with good recovery did not differ between groups. We could not verify the protective effect of hypothermia observed in post hoc analysis of National Acute Brain Injury Study: Hypothermia I and II data; however, our results align with the recent POLAR-RCT and Eurotherm3235 trials. The international POLAR-RCT study which investigated prophylactic hypothermia in acute severe TBI demonstrated no difference in favorable outcomes between hypothermia (48.8%) and normothermia (49.1%) patients (absolute risk difference, − 0.4, 95% CI, − 9.4 to 8.7; unadjusted relative risk with hypothermia, 0.99, 95% CI, 0.82–1.19, *p* = 0.94) [34]. The Eurotherm3235 trial evaluated the effect of hypothermia on elevated ICP and 6-month GOSE outcome after TBI. Eurotherm3235 ended early on recommendation of their DSMB. Final analysis of a dichotomized GOSE favored standard care (OR 1.74, 95% CI 1.09–2.77) [13].

### Circulating Levels of GFAP and UCH-L1

GFAP is an intermediate filament cytoskeletal protein found primarily in astrocytes [35]. GFAP is released into the circulation after TBI and early elevated GFAP levels in the plasma are predictors of poor outcome [30]. Consistent with this, we demonstrated higher GFAP levels in patients with poor outcome. UCH-L1, an abundant protein expressed in neurons, is involved in repair of injured axons and neurons [36]. We did not detect an association between UCH-L1 levels and outcome. Other studies have supported GFAP as being superior to UCH-L1 at predicting outcome [37–39]. Circulating GFAP and UCH-L1 measured together are biomarkers for severe TBI [20, 40]. Consistent with previously published studies, our patients’ plasma levels of GFAP and UCH-L1 were elevated within 6 h after injury and decreased to levels comparable to healthy comparators by T3 [19, 20].

To examine the effect of induced hypothermia on biomarker levels, we analyzed the plasma concentration over time and did not see a group difference in GFAP or UCH-L1 levels. Contrary to our findings, rodent TBI models have demonstrated a reduction in UCH-L1 levels with hypothermia treatment [41, 42]. However, after cardiac arrest UCH-L1 levels did not differ between comatose patients with cardiac arrest maintained at 36 °C compared with those maintained at 33 °C [43]. Mondello et al. [44] reported higher serum UCH-L1 levels in diffuse TBI compared with patients with mass lesions (*p* = 0.01) and higher GFAP levels in patients with mass lesions than those with diffuse injury (*p* = 0.006). We do not have evidence for differing degrees of neuronal injury between our temperature groups. The hematoma size and amount of shift were similar and diffuse axonal injury was absent in comparable proportions of patients. However, it is plausible that there were differing degrees of diffuse injury between groups. It is also plausible that both the hypothermia and protecting from fever with controlled normothermia may have deterred pathological processes associated with diffuse injury.

### Limitations

Our enrollment was slower than expected and resulted in an insufficient sample size to meet our study objectives. Review of our enrollment criteria indicate the key explanations for problems enrolling. Age limits (37%) and lack of SDH or planned surgical evacuation (29%) were the leading exclusion factors. Older age was associated with poorer outcome and more complications in patients with TBI hypothermia [14] and after acute SDH [45]. Younger ages (16–21 years) were excluded from our study because they are classified as pediatric patients by the United States Food and Drug Administration. The Brain Trauma Foundation Guidelines on surgical management of acute SDH recommend surgical evacuation for (1) SDH with thickness > 10 mm or midline shift > 5 mm and (2) on patients with SDH with GCS < 9 or neurodeterioration of 2 or more points on the GCS and/or asymmetric or fixed and dilated pupils and/or ICP > 20 mmHg [46]. Patients who do not meet these criteria may be observed closely and managed non-operatively. If the patient neurologically deteriorates and/or a repeat head computed tomography indicates that the brain injury has worsened, the patient will receive delayed surgery within 2–4 h of clinical deterioration. Guidelines indicate that surgery performed 2–4 h after clinical deterioration result in superior outcome compared to delayed surgery.

We have learned that a large proportion of patients with acute SDH do not meet Brain Trauma Guidelines criteria for surgical intervention. A retrospective chart review revealed that 646 of 869 (74.3%) of patients with acute traumatic SDH at a major level I trauma center were managed without surgical intervention. Only 6.5% of these patients required a delayed surgical evacuation at a median of 9.5 days after injury. GOS at discharge was good in 77% of the non-operatively managed patients [47]. Our criteria required that patients receive surgery within 6 h of injury, and those patients requiring surgery outside of this window would have been excluded.

More than 15% of patients with SDH were excluded because they had non-survivable injuries, conditions contraindicating hypothermia or consent could not be obtained. Our enrollment criteria, based on previous therapeutic hypothermia studies, while restrictive were necessary for patient safety.

The study outcome was a general functional outcome, GOSE at 6 months after injury. It is possible that a more specific measure of cognitive function may have identified a treatment effect. GOSE is the current functional outcome standard in TBI studies. Our findings may not be generalizable to other centers with different management protocols. However, this was a multicenter, pragmatic trial with protocols based on published recommendations for methods of therapeutic hypothermia. We compared therapeutic hypothermia with controlled normothermia. In previous studies [14–16, 22], standard care normothermia was not controlled with a device. Controlled normothermia may have resulted in a smaller variation in the first 48 h of the normothermia temperature range in our study and thus a potentially protective effect from early fever. A meta-analysis of 39 studies including 14,431 patients indicated fever after neurological injury (traumatic, hemorrhagic, or ischaemic) is associated with worse outcome [48]. The future of targeted temperature management in TBI may focus on tightly controlled normothermia.

## Conclusions

This randomized trial of hypothermia after acute SDH was terminated due to a low predictive probability of meeting the study objectives. At the interim futility analysis there was no difference identified between temperature groups in functional outcome. While the technology for therapeutic hypothermia has advanced considerably over the past 25 years facilitating ease of use and good temperature control, the small percentage of patients who met enrollment criteria may be indicative of the limited possibilities for further study in acute SDH. Hypothermia did not affect circulating levels of GFAP and UCH-L1. Elevated plasma levels of GFAP and UCH-L1 within the first 6 h of TBI support the use of these proteins as biomarkers for TBI with SDH.

## Supplementary Information

Below is the link to the electronic supplementary material.

Supplementary Fig. 1 Clinical parameters. This trial was a multicenter, randomized, controlled, pragmatic study to determine the effect of hypothermia on patients with acute traumatic subdural hematoma (SDH) who required emergent evacuation of their hematomas. Controlled temperature management of hypothermia (core temperature of 35 °C prior to dura opening followed by 33 °C for 48 hours) was compared to normothermia (37 °C). All centers used the Brain Trauma Foundation Guidelines and were experienced in TBI care and the application of hypothermia. However, because differences in management could influence results, we performed a post hoc analysis on several clinical parameters, intracranial pressure (ICP), mean arterial blood pressure (MAP), blood gas, and blood glucose levels. Daily high and low values of these parameters were collected prospectively for 8 days. (A) Summary data showing high and low mean ICP (mm Hg) are binned by treatment group; (B) high and low MAP (mm Hg) binned by treatment group; and (C) mean high and low partial pressure of carbon dioxide (PaCO2, (mm Hg)) levels by treatment group. (D) Mean daily serum glucose (mg/dL) values by treatment group. No group differences were observed in these variables (TIF 139461 kb)

Supplementary Content 2 Consort Checklist (DOC 219 kb)

## Declarations

### Clinical trial registration

This trial was registered in ClinicalTrials.gov as NCT02064959, and UMIN000014863 in the Japanese University hospital Medical Information Network (UMIN) Clinical Trials Registry (UMIN-CTR)https://clinicaltrials.gov/ct2/show/NCT02064959?term=Hypothermia%2C+subdural+hematoma&draw=2&rank=1.
